# Adherence to epidemiological measures and related knowledge and attitudes during the coronavirus disease 2019 epidemic in Croatia: a cross-sectional study

**DOI:** 10.3325/cmj.2020.61.508

**Published:** 2020-12

**Authors:** Mario Marendić, Ivana Bokan, Ivan Buljan, Paula Dominiković, Ružica Suton, Ivana Kolčić

**Affiliations:** 1 Department of Nursing, University Department of Health Studies, University of Split, Split, Croatia; 2 Department of Nursing, Health School Split, Split, Croatia; 3 Department for Research in Biomedicine and Health, University of Split School of Medicine, Split, Croatia; 4 Undergraduate student, University Department of Health Studies, University of Split, Split, Croatia; 5 Graduate student, Department of Neurology, University Department of Health Studies, University Hospital Center Split, Split, Croatia; 6 Department of Public Health, University of Split School of Medicine, Split, Croatia

## Abstract

**Aim:**

To assess the use of personal protective equipment (PPE) and related knowledge and attitudes during the coronavirus disease 2019 epidemic in Croatia.

**Methods:**

The online survey, conducted on social media in May 2020, yielded 1393 responses across the country (66% from the Adriatic area). The questionnaire consisted of socio-demographic questions and questions on the knowledge, attitudes, and behaviors related to PPE use. The χ^2^ test, *t* test, and multivariate logistic regression were used in data analysis.

**Results:**

As many as 84.0% of participants reported the compliance with social distancing measures, while 52.8% reported using PPE (mask and/or gloves) when shopping or visiting friends and family. Participants demonstrated good knowledge (mean of 10.4 [95% CI 10.3-10.4] correct answers out of 13 questions) and neutral to moderately positive attitude about PPE use (mean of 36.6 [36.1-37.1] out of 50 points). Participants with higher education, women, and health care workers had a greater probability for having a high knowledge score. Women, older individuals, public transport users, people with more positive PPE use attitude, and those who complied with social distancing had a higher probability of PPE use, while health care workers and highly educated participants had a reduced probability of PPE use in public.

**Conclusions:**

Croatians had good knowledge and neutral to moderately positive attitudes about PPE use. Nevertheless, health authorities need to promote positive attitudes about PPE use in order to retain trust and compliance with epidemiological measures.

The coronavirus disease 2019 pandemic is one of the major health and economic crises in modern history (1). The novel coronavirus (SARS-CoV-2), first recognized in the Chinese city of Wuhan in December 2019, has rapidly spread worldwide causing respiratory infections in humans (2). On January 30, 2020, the World Health Organization (WHO) declared an international public health emergency with an aim to establish international collaboration and control the outbreak (3). Among the main WHO recommendations for virus spread prevention were early detection and isolation of sick persons, contact tracing, physical distancing measures, and the use of personal protective equipment (PPE) (4).

This pandemic has become the topic of intensive research, with an unprecedented number of articles published in a very short period (5). Many studies have investigated the compliance with epidemiological measures and protective equipment use. Studies conducted worldwide among general population and health care workers between March and July 2020 have shown high knowledge and positive attitudes on preventive practices but also variable actual practices (6).

The first case of SARS-CoV-2 infection in Croatia was confirmed on February 25, 2020 (7). By the end of March, Croatia had introduced some of the strictest epidemiological measures in the world (8,9), such as banning public gatherings and sports events, closing of restaurants and non-essential shops, and border closing (lockdown period). Educational institutions switched to online teaching on March 16 (10). Epidemiological measures lasted 30 days, after which the Civil Protection Directorate of the Ministry of the Interior of Croatia (CPD) started restriction lifting in three phases (Supplementary Figure 1(supplementary figure 1)) (11,12).

From February 25 to May 25, the CPD held a daily press conference, at which they issued epidemic-related information, such as the number of new COVID-19 cases, and announced new restrictions.

Knowledge and attitudes are important determinants of behavior (13-15), which is of special importance in pandemic outbreaks. The aim of our study was to investigate the level of knowledge and attitudes about PPE use in Croatia during a spring period characterized by a declining epidemic curve. Additionally, we aimed to assess the characteristics associated with the adherence to epidemiological measures in the general population, and to compare knowledge, attitudes, and behaviors between health care and non-health care workers.

## METHODS

### Study design and setting

This cross-sectional study was conducted in May 2020, after the third stage of loosening of lockdown restrictions in Croatia. During data collection period, from May 11 to May 25, there were 57 new cases of COVID-19 in Croatia (16). This was a nation-wide study, with 21 Croatian counties being categorized into three zones: Adriatic Croatia, Central and Eastern Croatia, and North-Western Croatia.

Data were collected via an online anonymous questionnaire (Google form), the completion of which took on average 8 minutes. Before answering the questionnaire, participants were informed about the purpose and aims of the study, and were asked to confirm their consent to participate.

The convenience sampling method was used. We shared the questionnaire link on personal Facebook walls, Facebook groups of the general population, and health care professionals’ groups, and refreshed the posts after seven days. The questionnaire link was sent only once via WhatsApp, without reminders. The inclusion criteria were age >18 years and Croatian citizenship, leading to the exclusion of 9 underage participants. The study was approved by the Ethics Committee of the University of Split, Department of Health Studies (2181-228-07-20-0002).

### Questionnaire

We used a newly constructed questionnaire, divided into three sections. The first section consisted of 16 questions related to the participant’s socio-demographic characteristics and behavior. General data included sex, age, marital status, education degree, health care worker (no/yes, including doctors, nurses, pharmacists, radiological technologists, laboratory engineers, etc), place of residence (urban, semi-urban, village), transportation mode (use of private vehicle, public transport, or walking), PPE use (mask, gloves), and health status (yes/no for presence of any chronic disease). Additionally, we inquired about the participants' perceived need for additional education regarding PPE use. The second section consisted of 13 questions intended to assess the knowledge about appropriate PPE use. Knowledge-related questions were based on the recommendations of the Croatian Institute of Public Health (CIPH) (17-20), which were made in accordance with the WHO recommendations (21-24). Correct answers are shown in the Supplementary Table 1(supplementary table 1). The third section consisted of 10 statements related to the attitudes about PPE use. Participants rated each statement on a 5-point Likert-type scale (1 – strongly disagree; 2 – somewhat disagree; 3 – neither agree nor disagree; 4 – somewhat agree; 5 – strongly agree). The total score was calculated by summing up the points obtained on each answer, while the maximum score of 50 points represented the most positive attitude.

### Data analysis

The normality of distribution was assessed with the Kolmogorov-Smirnov test. Categorical variables are presented as frequencies and percentages, while numerical variables are presented as means with 95% confidence intervals (CI) or medians with interquartile range. The factor analysis (Promax method) was used to determine the factor structure of the questionnaire on attitudes about PPE use, and detected only one underlying factor (Supplementary Table 2(supplementary table 2)). Additionally, the reliability was estimated using the Cronbach’s alpha coefficient, which was 0.87 (95% CI, 0.86-0.88).

The differences between health care and non-health care workers were assessed using the χ^2^ test for categorical variables and the *t* test for numerical variables. Logistic regression was used to identify the characteristics associated with high knowledge score (≥12 correct answers out of 13 questions) and PPE use during transportation, walking, shopping, or visiting family and friends. Each of the three logistic regression models included the following independent variables: sex, age, education (three categories: high school or less; university level including both undergraduate and graduate studies; master’s degree or PhD), urbanization level (urban, semi-urban, village), health care worker status (yes/no), chronic disease (present/absent), and total attitude score. Additionally, two regression models with PPE use as dependent variables included the previously mentioned independent variables, alongside with transportation type, total knowledge score, and complying with social distancing (yes/no). We also presented Cox & Snell R^2^ regression result. All analyses were performed with JASP statistical software, v.12.0.0. (JASP Team, 2020), with the level of significance set at *P* < 0.05.

## RESULTS

The study enrolled 1393 participants (median age 34 years, IQR 24-43), mainly women (84.4%), 65.5% of whom lived in the Adriatic part of Croatia (Table 1). The majority of participants were well educated (only 33.9% of participants had completed high school or less), employed (68.6%), urban residents (64.6%), and lived in a family with three or more members (76.3%) (Table 1). Healthcare and non-health care workers differed in sex composition (90.1% of health care workers were women), regional distribution, education level, and employment (Table 1).

**Table 1:** Demographic characteristics of the overall sample and comparison between health care workers and non-health care workers (data presented as absolute numbers and percentages)

	Overall sample (N = 1393)	Non-health care workers (n = 757)	Healthcare workers (n = 636)	P^*^
Sex				
female	1175 (84.4)	602 (79.5)	573 (90.1)	<0.001
male	218 (15.6)	155 (20.5)	63 (9.9)	
Age (median, interquartile range)	34 (24-43)	32 (23-44)	35 (25-42)	0.225
Region				
Adriatic Croatia	913 (65.5)	522 (69.0)	391 (61.5)	0.005
Central and Eastern Croatia	162 (11.6)	72 (0.5)	90 (14.2)	
North-Western Croatia	318 (22.8)	163 (21.5)	155 (24.4)	
Education degree				
elementary school	3 (0.2)	3 (0.4)	0 (0.0)	<0.001
high school	470 (33.7)	267 (35.3)	203 (31.9)	
undergraduate study	385 (27.6)	151 (19.9)	234 (36.8)	
graduate study	346 (24.8)	244 (32.2)	102 (16.0)	
master's degree	137 (9.8)	71 (9.4)	66 (10.4)	
postgraduate studies	52 (3.7)	21 (2.8)	31 (4.9)	
Employment				
unemployed	113 (8.1)	100 (13.2)	13 (2.0)	<0.001
high school student	45 (3.2)	18 (2.4)	27 (4.2)	
university student	253 (18.2)	171 (22.6)	82 (12.9)	
retired	25 (1.8)	20 (2.6)	5 (0.8)	
employed	956 (68.6)	448 (59.2)	508 (79.9)	
Living				
with a partner	221 (15.9)	119 (15.7)	102 (16.0)	0.854
alone	109 (7.8)	62 (8.2)	47 (7.4)	
in a family with three or more members	1063 (76.3)	576 (76.1)	487 (76.6)	
Current residence				
urban	900 (64.6)	477 (63.0)	423 (66.5)	0.082
semi-urban	225 (16.2)	118 (15.6)	107 (16.8)	
village	268 (19.2)	162 (21.4)	106 (16.7)	

*χ^2^ test.

The majority of the participants used their own car for transportation (73.7%) (Table 2). Just above one quarter of the participants (28.8%) stated that they used PPE during transportation and walking (31.8% non-health care workers vs 25.2% health care workers) (Table 2). Less than half of the participants (47.2%) stated that they did not use PPE when shopping or visiting family and friends. In such occasions, health care workers, compared with non-health care workers, more frequently wore a mask (31.3% vs 22.2%), and less frequently wore only gloves (1.7% vs 9.2%). The compliance with the CPD measures was reported by 59.7% of participants, and the compliance with social distancing by 84.0% of participants (Table 2).

**Table 2:** Coronavirus disease 2019-related behavior in the overall sample and the comparison between non-health care and health care workers (data presented as absolute numbers and percentages)*

		Overall sample (N = 1393)	Non-health care worker (n = 757)	Healthcare worker (n = 636)	*P^†^*
**Do you use any transport?**					
I'm walking		228 (16.4)	153 (20.2)	75 (11.8)	<0.001
I use public transport		138 (9.9)	82 (10.8)	56 (8.8)	
I use a personal vehicle		1027 (73.7)	522 (69.0)	505 (79.4)	
**Using of protective equipment (mask or gloves) while walking, using public city transport, or using a personal vehicle**		401 (28.8)	241 (31.8)	160 (25.2)	0.006
**When leaving the house to go shopping or to visit family and friends, I use**					
mask		367 (26.3)	168 (22.2)	199 (31.3)	<0.001
gloves		81 (5.8)	70 (9.2)	11 (1.7)	
mask and gloves		288 (20.7)	166 (21.9)	122 (19.2)	
none of the above		657 (47.2)	353 (46.6)	304 (47.8)	
**Compliance with the measures recommended by the Civil Protection Directorate**					
I don’t adhere to it at all		18 (1.3)	13 (1.7)	5 (0.8)	0.069
I adhere to it occasionally		200 (14.4)	122 (16.1)	78 (12.3)	
I adhere to most measures		344 (24.7)	178 (23.5)	166 (12.3)	
I adhere to all measures		831 (59.7)	444 (58.7)	387 (60.8)	
**Compliance with the measure of social distance**		1170 (84.0)	627 (82.8)	543 (85.4)	0.196
**I recovered from COVID-19 infection**					
yes		10 (0.7)	3 (0.4)	7 (1.1)	0.216
no		965 (69.3)	533 (70.4)	432 (67.9)	
don't know		418 (30.0)	221 (29.2)	197 (31.0)	
**I've been in contact with a person who recovered from COVID-19 infection**					
yes		126 (9.0)	14 (1.8)	112 (17.6)	<0.001
no		853 (61.2)	532 (70.3)	321 (50.5)	
don't know		414 (29.7)	211 (27.9)	203 (31.9)	
**Chronic disease present**		188 (13.5)	92 (12.2)	96 (15.1)	0.110
**Sources of information on protection from COVID-19**					
media articles on the internet		105 (7.5)	78 (10.3)	27 (4.3)	<0.001
internet in general		370 (26.6)	240 (31.7)	130 (20.4)	
website CIPH		202 (14.5)	42 (5.5)	160 (25.2)	
website of the Government of the Republic of Croatia		101 (7.3)	42 (5.5)	59 (9.3)	
I don't think about it		90 (6.5)	51 (6.8)	39 (6.1)	
newspaper		3 (0.2)	3 (0.4)	0 (0.0)	
through a friend		12 (0.9)	7 (1.0)	5 (0.8)	
TV (CPD, news)		429 (30.8)	264 (34.8)	165 (26.0)	
scientific articles		81 (5.8)	30 (4.0)	51 (8.0)	
**Self-perceived need of additional education on the use of personal protective equipment**					
yes	107 (7.7)		64 (8.5)	43 (6.8)	0.016
no	1200 (86.1)		635 (83.9)	565 (88.8)	
don't know	86 (6.2)		58 (7.7)	28 (4.4)	

*CPD – Civil Protection Directorate; CIPH – Croatian Institute of Public Health.

†χ^2^ test.

The majority of participants had not been diagnosed with COVID-19 and they had not been in contact with infected people. The prevalence of any chronic disease in the sample was 13.5% (Table 2).

The most common sources of information on protective measures against COVID-19 were TV news and CPD press conferences (30.8%) and internet in general (26.6%), while 14.5% of participants used the official CIPH website and 7.3% used the official website of the Croatian government. Only 7.7% of participants stated they needed additional education regarding PPE use (Table 2).

Participants mostly gave correct answers on questions related to PPE use (Table 3). On average, health care workers replied correctly to 10.6 questions and non-health care workers to 10.2 out of 13 questions (*P* < 0.001). Compared with non-health care workers, health care workers more frequently correctly answered the questions related to masks wearing and hygienic hand washing (Table 3). Non-health care workers more frequently correctly answered the questions on gloves disinfection and PPE use while driving in a personal vehicle with persons other than household members (Table 3).

**Table 3:** The proportion of correct answers on the questions related to the use of personal protective equipment in the overall sample and comparison between health care and non-health care workers (data presented as absolute numbers and percentages)

Questions	Correct answers	Overall sample (N = 1393)	Non-health care workers (n = 757)	Healthcare workers (n = 636)	P*
I need to replace the mask when it gets wet.	True	1247 (89.5)	688 (90.9)	559 (87.9)	0.069
It is allowed to disinfect gloves with alcohol to keep them clean during use.	True	835 (59.9)	482 (63.7)	353 (55.5)	0.002
Gloves protect against infection caused by contact.	False	283 (20.3)	155 (20.5)	128 (20.1)	0.872
Personal protective equipment should be used when driving in a personal vehicle with a person other than your household members.	True	1165 (83.6)	653 (86.3)	512 (80.5)	0.004
Gloves and masks may be used repeatedly.	False	1284 (92.2)	667 (88.1)	617 (97.0)	<0.001
Proper hand washing is one of the prevention measures from coronavirus infection.	True	1366 (98.1)	739 (97.6)	627 (98.6)	0.194
The mask protects against respiratory infection.	True	984 (70.6)	484 (63.9)	500 (78.6)	<0.001
Personal protective equipment should be used when driving a personal vehicle (when you are alone in the car).	False	1314 (94.3)	700 (92.5)	614 (96.5)	0.001
Cotton masks have the same protection effectiveness as the standard surgical masks.	False	1140 (81.8)	587 (77.5)	553 (86.9)	<0.001
When I put on the mask, it should cover both the nose and the mouth.	True	1378 (98.9)	746 (98.5)	632 (99.4)	0.138
I should use a mask and gloves every time I leave the house.	False	791 (56.8)	375 (49.5)	416 (65.4)	<0.001
It is necessary to perform hygienic hand washing after removing protective gloves.	True	1330 (95.5)	708 (93.5)	622 (97.8)	<0.001
Personal protective equipment should be used when using public transport.	True	1336 (95.9)	729 (96.3)	607 (95.4)	0.419
Total (Mean, 95% confidence interval)^†^		10.4 (10.3-10.4)	10.2 (10.1-10.2)	10.6 (10.5-10.7)	<0.001

*χ^2^.

† *t* test for independent samples.

The total average score on the part of the questionnaire regarding PPE-related attitudes was 36.6/50 points, indicating a neutral to moderately positive attitude, with health care workers having a higher score than non-health care workers (37.5 vs 35.9; *P* = 0.002) (Table 4). The lowest attitude score was recorded for PPE use during shopping, for buying enough PPE regardless of the price, and for feeling safe while wearing mask or gloves. The highest positive score was recorded for advising persons with chronic diseases to wear PPE, higher risk for infection in health care professionals, compliance with social distancing as one of the best prevention measures, and the importance of hygienic hand washing (Table 4).

**Table 4:** Attitudes about the use of personal protective equipment against coronavirus disease 2019 (COVID-19) in the overall sample and comparison between health care and non-health care workers (data are presented as mean, 95% confidence interval)

Items	Overall sample (N = 1393)	Non-health care workers (n = 757)	Healthcare workers (n = 636)	P*
I consider that hygienic hand washing can reduce the risk of infection with the novel coronavirus.	4.3 (4.3-4.4)	4.3 (4.2-4.3)	4.4 (4.3-4.5)	0.027
I maintain social distance and I use protective equipment (masks or gloves).	3.4 (3.3-3.5)	3.3 (3.2-3.4)	3.6 (3.4-3.6)	0.002
Gloves and a mask protect against the disease caused by the novel coronavirus.	3.5 (3.4-3.5)	3.4 (3.3-3.5)	3.6 (3.5-3.7)	0.046
Regardless of the price, I always buy enough protective equipment.	3.1 (3.0-3.1)	3.0 (2.9-3.1)	3.2 (3.0-3.3)	0.049
Healthcare professionals have a higher risk for infection with COVID-19.	4.4 (4.3-4.4)	4.3 (4.2-4.4)	4.4 (4.3-4.5)	0.207
I feel safe when I wear a mask or gloves.	3.2 (3.1-3.3)	3.2 (3.1-3.3)	3.2 (3.1-3.3)	0.744
Without protective equipment, I am not going to shop for groceries and hygiene products.	2.9 (2.9-3.0)	3.0 (2.9-3.1)	2.9 (2.7-3.0)	0.204
Protective equipment is extremely important in my environment (business, family etc.).	3.3 (3.2-3.3)	3.0 (3.0-3.1)	3.6 (3.5-3.7)	<0.001
Compliance with social distancing is one of the best measures to prevent the spread of infection.	4.3 (4.2-4.3)	4.2 (4.1-4.3)	4.4 (4.3-4.4)	0.010
Persons suffering from chronic disease should be advised to wear protective equipment	4.4 (4.4-4.5)	4.4 (4.3-4.9)	4.5 (4.4-4.6)	0.119
Total score (sum of answers)	36.6 (36.1-37.1)	35.9 (35.3-36.6)	37.5 (36.7-38.2)	0.002

*t-test for independent samples.

The logistic regression model showed several characteristics to be associated with high knowledge scores about PPE use (Table 5). Women had higher probability of high knowledge score compared with men (OR = 1.68; 95% CI 1.05-2.70; *P* = 0.032), as well as participants with higher education level compared with participants with high school education. Healthcare workers had 57% higher probability of high knowledge score compared with non-health care workers (OR = 1.57; 95% CI 1.17-2.10; *P* = 0.003) (Table 5). Age, urbanization level, chronic disease presence, and PPE use attitudes were not associated with high PPE knowledge score (Table 5). The model explained 2.8% of the variance.

**Table 5:** Logistic regression model of characteristics associated with high knowledge scores (≥12) about the use of personal protective equipment (PPE) against coronavirus disease 2019

Variables	Odds ratio (95% confidence interval); *P*
Women (men are referent group)	1.68 (1.05-2.70); 0.032
Age (years)	1.01 (0.99-1.02); 0.130
Education (high school is referent)	
university	1.66 (1.18-2.35); 0.004
MSc or PhD	1.75 (1.10-2.79); 0.019
Healthcare workers (non-health care workers are referent)	1.57 (1.17-2.10); 0.003
Urbanization level (urban is referent)	
semi-urban	0.99 (0.66-1.49); 0.966
village	0.90 (0.61-1.34); 0.603
Chronic disease present	1.12 (0.74-1.70); 0.593
PPE use attitudes (total score)	1.01 (0.99-1.03); 0.095

Finally, we assessed the association between participants’ characteristics and PPE use (Table 6). Female sex (OR = 1.56, 95% CI 1.05-2.32; *P* = 0.029), older age (OR = 1.02, 95% CI 1.01-1.03; *P* = 0.003), use of public transport (OR = 4.80, 95% CI 3.11-7.41; *P* < 0.001), walking (OR = 1.60, 95% CI 1.12-2.29; *P* = 0.010), more positive PPE use attitudes (OR = 1.11, 95% CI 1.09-1.13; *P* < 0.001), and compliance with social distancing (OR = 2.88, 95% CI 1.72-4.84; *P* < 0.001) were associated with a higher probability for PPE use during transportation, while chronic disease presence was marginally insignificant (OR = 1.45, 95% CI 0.98-2.14; *P* = 0.060). On the other hand, participants with higher education level, health care workers, and people with higher PPE knowledge score had a lower probability for PPE use during transportation and walking (Table 6). These variables explained 28.2% of the variance (Cox & Snell R^2^).

**Table 6:** Logistic regression model of characteristics associated with the use of personal protective equipment (PPE) against coronavirus disease 2019 (COVID-19)

Variables	Use of PPE during transportation and walking	Use of PPE when shopping or visiting family and friends
	**OR (95% CI); *P***	**OR (95% CI); *P***
Women (men are referent group)	1.56 (1.05-2.32); 0.029	1.37 (0.96-1.94); 0.083
Age (years)	1.02 (1.01-1.03); 0.003	1.03 (1.01-1.04); <0.001
Education (high school is referent)		
university	0.71 (0.53-0.95); 0.021	0.77 (0.58-1.02); 0.066
MSc or PhD	0.26 (0.40-0.95); 0.027	0.55 (0.37-0.83); 0.004
Healthcare workers (non-health care workers are referent)	0.64 (0.49-0.84); 0.001	0.74 (0.58-0.96); 0.023
Urbanization level (urban is referent)		
semi-urban	1.25 (0.87-1.80); 0.229	1.44 (1.01-2.04); 0.043
village	1.01 (0.71-1.44); 0.938	1.05 (0.76-1.46); 0.757
Transportation (personal vehicle is referent)		
public transport	4.80 (3.11-7.41); <0.001	0.97 (0.63-1.49); 0.897
walking	1.60 (1.12-2.29); 0.010	0.85 (0.60-1.20); 0.350
Chronic disease present	1.45 (0.98-2.14); 0.060	1.12 (0.76-1.65); 0.575
PPE use attitudes (total score)	1.11 (1.09-1.13); <0.001	1.12 (1.10-1.14); <0.001
PPE knowledge (total score)	0.84 (0.75-0.94); 0.002	0.93 (0.84-1.04); 0.199
Compliance with social distancing	2.88 (1.72-4.84); <0.001	3.50 (2.36-5.19); <0.001

*OR – odds ratio; CI – confidence interval.

A greater probability for PPE use when shopping or visiting family and friends was observed in older participants, less educated participants, non-health care workers, individuals from semi-urban areas, participants with more positive attitudes about PPE use, and in those who complied with social distancing. These variables explained 33.9% of the variance (Table 6).

## DISCUSSION

Similarly to findings from other countries, our study showed that Croatian citizens had good knowledge and neutral to moderately positive attitudes about the use of PPE for COVID-19 (13-15,25-29). During the post-lockdown period in spring 2020, the majority of participants (84%) reported to adhere to social distancing, and 52.8% reported wearing mask and/or gloves while shopping or visiting family and friends. Only a quarter of respondents used PPE during transport or walking, which can be explained by the fact that many people used personal vehicles for transportation (73.7%). Another Croatian study showed a very high restrictions compliance during the lockdown (96%), especially among women and participants with higher education (30).

Our results showed that women, participants with higher education, and health care workers had greater probability for having high knowledge about PPE use. Similarly, in a study from China, women, unmarried people, health care workers, and people with higher education levels displayed higher knowledge (29).

Given the high knowledge about PPE use in our sample, we wanted to know whether knowledge and attitudes about PPE use were associated with the actual PPE use. Expectedly, female sex, older age, and public transport use were associated with a higher probability for PPE use. However, a surprising finding was that positive attitude was a more important predictor for PPE use than knowledge. Contrary to our expectations, higher PPE knowledge was not associated with the actual PPE use during shopping and visiting family and friends, and it was even negatively associated with PPE use during transportation or walking. This is not a novel finding, even in the domain of COVID-19 research. For example, Bates et al concluded that “knowledge about COVID-19 is insufficient to prompt behavioral change” (15). We also showed that preventive behaviors tend to cluster together, since the compliance with social distancing was a strong predictor for PPE use.

Another interesting finding was that health care workers had lower probability of PPE use in public, when other confounding factors were taken into account. This was despite their higher knowledge and more positive attitude about PPE use, alongside habitual PPE use in their workplace setting. A comparable result was reported in a study from Pakistan, where health studies students showed good knowledge and positive attitudes, but unsatisfactory preventive practices (27). Similar findings were also shown in pharmacy students from Egypt (31). Although health care workers commonly use PPE in their workplace, they should be encouraged to wear it in public, thus setting a good example. However, it is noteworthy that during the study period in Croatia PPE use while shopping and commuting was not mandatory (32,33).

The main source of information about coronavirus during spring 2020 were TV news and CPD conferences. The secondary source of information for non-health care workers was the internet, while for health care workers it was the CIPH website, which is a professional and credible information source. As a matter of comparison, a Vietnamese study reported that health care workers predominately used social media to inform themselves about COVID-19 (34).

Tightening of measures in autumn due to increase in the number of the COVID-19 cases in Croatia was met by public protests, indicating a lack of the population's trust in the government and health care professionals. To regain trust, the authorities should boost positive attitudes about epidemiologic measures rather than just repeatedly stressing the importance of adherence to measures (15).

One of the study limitations is data collection through social media, but due to epidemic restrictions at the time of study execution this was the only available method. Surveys conducted via social media often fail to include men and older people (35). More precisely, this study enrolled mainly younger and well educated women, which may limit the generalizability of our findings. Next, the decline in the epidemic curve and loosening of the epidemiological measures might have affected the PPE use, which was not mandatory at the time of data collection. Most importantly, our sample is not country-representative, given that more than half of our respondents came from the Adriatic region of Croatia (65.5%). On the other hand, this is the first study to explore the important parameters influencing the epidemic curve, such as the knowledge and attitudes about PPE use, as well as the actual PPE use in Croatia. Studies on knowledge, attitudes, and adherence to epidemiological measures, especially PPE use, on the population level might be used to help public health authorities plan transmission control during the ongoing epidemic. For example, wearing masks and implementing policies requiring their use was proven effective in the mitigation of the SARS-CoV-2 spread (36).

Despite satisfactory adherence to epidemiological measures, especially social distancing, during springtime, and good knowledge about PPE use, our respondents displayed neutral to only moderately positive attitudes about PPE use. This finding should be brought into the attention of health authorities, who should work toward raising positive attitudes and optimism in the population. These, along with giving unequivocal instructions, can help in keeping the citizens’ trust and compliance. Although health care workers expectedly displayed greater knowledge, they should be encouraged to use PPE outside their workplace too. Given the recent increase in new cases of COVID-19 in Croatia, knowledge, attitudes, and practices in the population should be continuously monitored in order to control disease transmission in the months to come.
