# Supplementary figures and images for: Adherence to epidemiological measures and related knowledge and attitudes during the coronavirus disease 2019 epidemic in Croatia: a cross-sectional study
Source: Croat Med J. 2020 Dec;61(6):508–17. doi: 10.3325/cmj.2020.61.508 (PMC7821367; doi:10.3325/cmj.2020.61.508)

**Supplementary Figure 1.** Three phases of loosening epidemiological measures (12, 13)

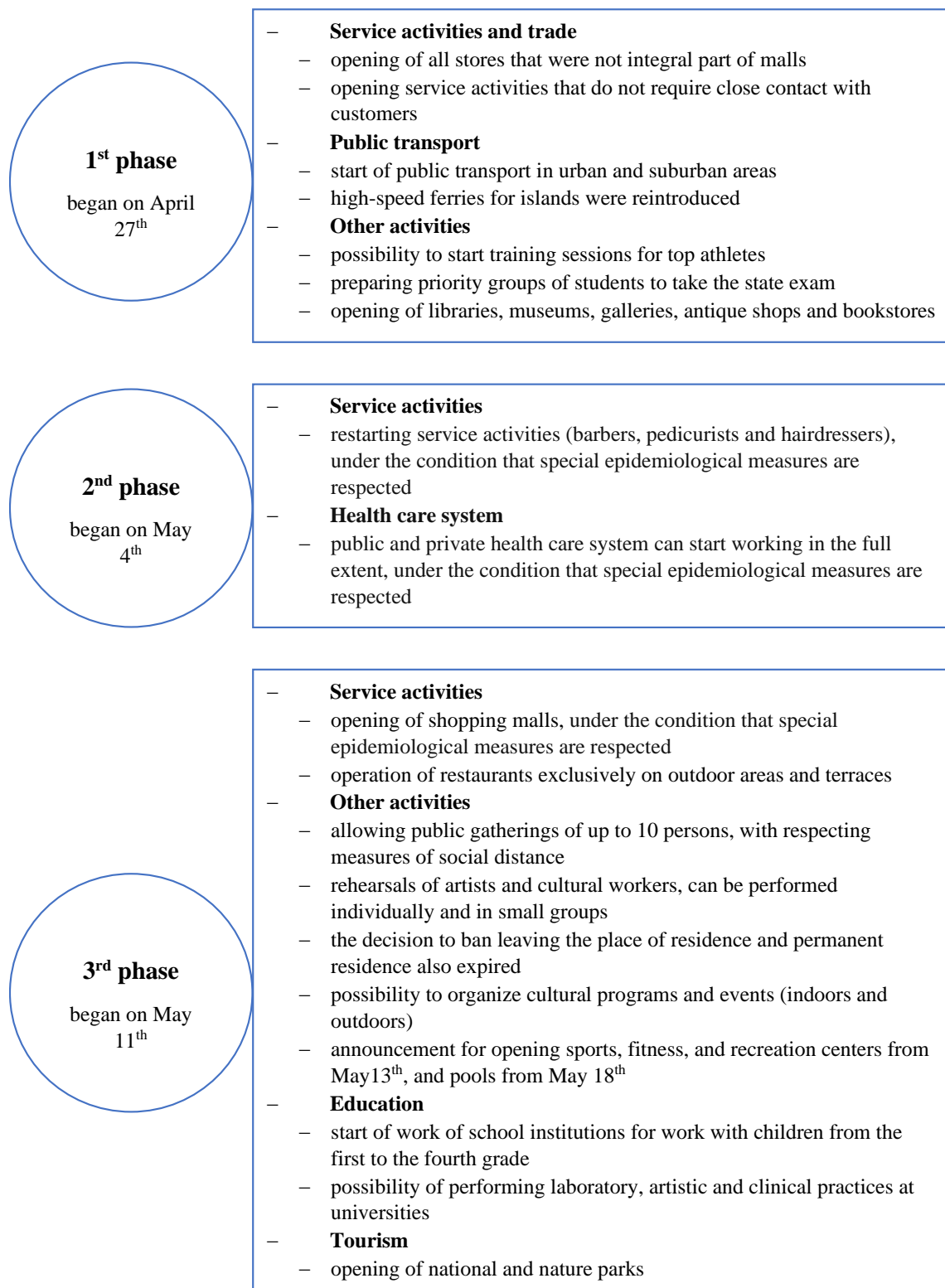

Supplement: Supplementary Figure 1 [file CroatMedJ_61_s001.pdf]
