# Supplementary material for: Adherence to epidemiological measures and related knowledge and attitudes during the coronavirus disease 2019 epidemic in Croatia: a cross-sectional study
Source: Croat Med J. 2020 Dec;61(6):508–17. doi: 10.3325/cmj.2020.61.508 (PMC7821367; doi:10.3325/cmj.2020.61.508)
Supplement: Supplementary Table 1 [file CroatMedJ_61_s002.pdf]

Supplemental table 1. Presentation of correct answers on knowledge questions according to Croatian Institute of Public Health (CIPH) (17-20).

No.	Questions	Correct answers	CIPH
**1.**	I need to replace the mask when it gets wet.	True	The moistened mask should be removed and replaced with a new, dry one.
**2.**	It is allowed to disinfect gloves with alcohol to keep them clean during use.	True	Controllers in contact with passengers should wear disposable masks and gloves, which must be disinfected or replaced after the tram is changed.
**3.**	Gloves protect against infection caused by contact.	False	Gloves give a false sense of security. If they don't change regularly, that wearing doesn't make sense, which means the virus needs to bring touching ears to contaminated gloves.
**4.**	Personal protective equipment should be used when driving in a personal vehicle with a person other than your household members.	True	If the driver's wearing of the mask does not interfere with safe driving, it is advisable to wear the mask while driving.
**5.**	Gloves and masks may be used repeatedly.	False	Gloves and masks may be used once.
**6.**	Proper hand washing is one of the prevention measures from coronavirus infection.	True	Washing your hands with soap and water is a satisfactory measure of protection.
**7.**	The mask protects against respiratory infection.	True	The physical barrier we create by wearing a mask can help reduce the spread of infection by providing us with protection from larger droplets that may contain the virus and preventing contact between contaminated hands and mucous membranes.
**8.**	Personal protective equipment should be used when driving a personal vehicle (when you are alone in the car).	False	There is no need to wear a mask when you are alone in the car.
**9.**	Cotton masks have the same protection effectiveness as the standard surgical masks.	False	Cotton masks slow down the flow of respiratory drops, while surgical masks protect others from the respiratory drops of the person wearing the mask.
**10.**	When I put on the mask, it should cover both the nose and the mouth.	True	The mask should cover the mouth, nose and part of the face.
**11.**	I should use a mask and gloves every time I leave the house.	False	Persons without respiratory symptoms do not need personal protective equipment.
**12.**	It is necessary to perform hygienic hand washing after removing protective gloves.	True	Wash your hands thoroughly with soap before and after applying protective equipment.
**13.**	Personal protective equipment should be used when using public transport.	True	Passengers are advised to use a mask while driving, if they do not mind breathing due to their health condition.
