# Supplementary material for: Adherence to epidemiological measures and related knowledge and attitudes during the coronavirus disease 2019 epidemic in Croatia: a cross-sectional study
Source: Croat Med J. 2020 Dec;61(6):508–17. doi: 10.3325/cmj.2020.61.508 (PMC7821367; doi:10.3325/cmj.2020.61.508)
Supplement: Supplementary Table 2 [file CroatMedJ_61_s003.pdf]

Supplemental table 2. Factor analysis of the items in the attitudes questionnaire towards personal protective equipment use

	Factor loadings	Item total correlation
I consider that hygienic hand washing can reduce the risk of infection with novel coronavirus.	0.592	0.531
I maintain social distance and I use protective equipment (masks or gloves).	0.716	0.672
Gloves and a mask protect against diseases caused by novel coronavirus.	0.622	0.578
Regardless of the price, I always buy enough protective equipment.	0.644	0.608
Healthcare professionals have a higher risk for infection with COVID-19.	0.594	0.538
I feel safe when I wear a mask or gloves.	0.676	0.639
Without protective equipment, I am not going to shop for groceries and hygiene products.	0.607	0.571
Protective equipment is extremely important in my environment (business, family etc.).	0.682	0.645
Compliance with social distance is one of the best measures to prevent the spread of infection.	0.691	0.632
Persons suffering from chronic disease should be advised to wear protective equipment	0.579	0.525
